# A *De Novo* case of autosomal dominant mitochondrial membrane protein‐associated neurodegeneration

**DOI:** 10.1002/mgg3.1706

**Published:** 2021-05-27

**Authors:** Stuart Fraser, Mary Koenig, Laura Farach, Pedro Mancias, Kate Mowrey

**Affiliations:** ^1^ Department of Pediatrics Division of Child and Adolescent Neurology McGovern Medical School University of Texas Health Science Center at Houston Houston Texas USA; ^2^ Department of Pediatrics Division of Medical Genetics McGovern Medical School University of Texas Health Science Center at Houston Houston Texas USA

**Keywords:** brain‐iron accumulation, clinical genetics, movement disorders, neurodegeneration

## Abstract

**Background:**

Mitochondrial membrane protein‐associated neurodegeneration (MPAN) is a genetic neurodegenerative condition previously thought to be inherited only in an autosomal recessive pattern through biallelic pathogenic variants in *C19orf12*. Recent evidence has proposed that MPAN can also follow autosomal dominant forms of inheritance. We present a case of a *de novo* pathogenic variant in *C19orf12* identified in a female with clinical features consistent with a diagnosis of MPAN, adding further evidence that the disease can be inherited in an autosomal dominant fashion.

**Methods:**

A 17‐year‐old Hispanic female was born to non‐consanguineous healthy parents. She developed progressive muscle weakness and dystonia beginning when she was 12 years old. Trio, whole‐exome sequencing with mitochondrial genome sequencing, and deletion/duplication analysis of both nuclear and mitochondrial genomes was performed in December 2019.

**Results:**

Whole‐exome sequencing analysis revealed a single *de novo* variant in *C19orf12*. The specific variant is c.256C>T (p.Q86X) located in exon 3.

**Conclusion:**

Our clinical report provides further clinical evidence that MPAN can be inherited in an autosomal dominant or recessive fashion. The patient's age of onset and clinical symptoms are very similar to the previous patient published with this specific variant as well as others with heterozygous pathogenic variants in *C19orf12* in Gregory et al. 2019. Our case report highlights the importance of considering both autosomal dominant and autosomal recessive version of MPAN with all patients demonstrating clinical features suggestive of MPAN.

## Graphical abstract

This article presents a case of a patient with a heterozygous mutation in C19orf12. This gene causes Mitochondrial Membrane Protein Associated Neurodegeneration (MPAN), and has previously been thought to be caused only by autosomal recessive modes of inheritance. We reference two similar publications identifying heterozygous cases of MPAN, and emphasize that our clinical report provides further evidence that MPAN can be caused by homozygous or heterozygous genetic changes in C19orf12.

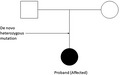

## 1 INTRODUCTION

Mitochondrial membrane protein‐associated neurodegeneration (MPAN [OMIM: 614298]) is part of a larger group of disorders known as neurodegeneration with brain iron accumulation (NBIA). The characteristic features of NBIA are iron accumulation in the basal ganglia in addition to various neurologic and psychiatric clinical features (Hogarth et al., 2013). Specifically, the iron accumulation in MPAN is noted to be in both the globus pallidus and substantia nigra (Hartig et al., 2011). MPAN can be further characterized by gait changes, progressive spasticity, dystonia, optic atrophy, muscle weakness, dysarthria, dysphagia, neuropsychiatric manifestations, and cognitive decline (Hogarth et al., 2013). The age of onset typically occurs in childhood to early adulthood. In comparison to other forms of NBIA, the disease progression of MPAN is slow with most patients surviving well into adulthood. The end stages of MPAN are characterized by loss of ambulation, weight loss, bladder and bowel incontinence, severe dementia, limited communication abilities, and stereotypic movements. Death typically occurs secondary to aspiration pneumonia (Gregory et al., 1993).

MPAN is estimated to have a prevalence of less than 1 in 1 million people (Gregory et al., 1993). Given the rarity of the condition, it has become better characterized and defined within the past decade (Schulte et al., 2013). The causative gene for MPAN is *C19orf12*. *C19orf12* is located at 19q12, spans 17 kb, and contains a total of 3 exons (Hartig et al., 2011). *C19orf12* encodes for a small transmembrane protein found in high concentrations in mitochondrial membranes and endoplasmic reticulum (Venco et al., 2015). Although the exact function of the protein is not yet understood, *C19orf12* is thought to play an important role in mitochondrial function and structure as well as fatty acid metabolism (Hartig et al., 2011).

Until recently, the inheritance for MPAN was thought to be entirely autosomal recessive (Hartig et al., 2011). However, Monfrini et al. 2018 published a case report of a patient with MPAN who had a novel heterozygous pathogenic variant, c.265_266delAT (p.M89Gfs*12), in the *C19orf12*. As the subject's pathogenic variant in this paper was *de novo*, the authors’ proposed a dominant‐negative mechanism leading to the clinical features in their patient (Monfrini et al., 2018). Shortly thereafter, Gregory et al. 2019 published 18 heterozygous cases of MPAN from 19 families from the Oregon Health & Science University (OSHU) International NBIA Repository. This publication provided additional clinical evidence that MPAN can manifest in the presence of a single. heterozygous pathogenic variant in *C19orf12*. In both publications, the individuals with the autosomal dominant form of MPAN were clinically indistinguishable from individuals with the autosomal recessive form. As a result, Gregory et al. 2019 echoed and further supported Monfrini et al. 2018’s hypothesis that nonsense truncating variants located in the final exon of *C19orf12* impairs the function of the wildtype protein, producing a dominant‐negative effect and leading to loss of function (Gregory et al., 2019).

Here, we provide a clinical report of an additional patient with suspected autosomal dominant MPAN who harbors the same pathogenic variant as Subject 227 in Gregory et al. 2019. Like subject 227, our patient became symptomatic at 12 years of age and suffered from optic atrophy, and progressive gait difficulties. Our case report is of significance as it can provide additional clinical evidence about the recently described autosomal dominant form of MPAN in hopes of impacting the clinical care of future patients with neurological disease.

## 2 CASE REPORT

### 2.1 Ethical compliance

Institutional review board approval was obtained for publication of this case report.

The proband was born after an uncomplicated pregnancy and had a routine delivery. Parents were non‐consanguineous. The mother was 38 years old at birth, and the father 37 years old. The proband was healthy throughout infancy and early childhood and met normal speech and motor milestones, though she was noted to be ‘clumsy’ by family members. At 10 years old she was noted to have poor vision and was referred to an ophthalmologist and diagnosed with optic atrophy. At 12 years of age, she began to complain of weakness in the lower extremities with frequent falls. She saw a private neurologist at 13 years of age, and an MRI of the brain without contrast was performed at the time and reported as normal. At 14 years of age, she began to complain of stiffness in the legs, ankles, and lower back exacerbated by sitting or lying down for long periods of time. The symptoms were progressive, and by 16 years of age, she was having several falls per day and had progressive dystonia in the lower extremities. She was referred for neurologic evaluation in 2018. At that time, her initial physical exam revealed gait difficulty and a positive Gowers sign.

After her neurologic evaluation, she was referred for basic laboratory studies followed by an electromyogram and nerve conduction study. Laboratory analysis revealed a mildly elevated creatine kinase (299 u/l, Reference Range: 12–191 µ/L), but a normal lactic acid (1.5 mmol/L, reference range 0.5–2.2 mmol/L) and a normal aldolase (6.4 µ/L, reference range 1.2–7.6 µ/L). The nerve conduction study revealed normal sensory and motor responses in the sural, ulnar, and tibial nerves. However, the electromyogram revealed fibrillations and positive sharp waves, consistent with axonal motor neuropathy. Due to her progressive symptoms, abnormal physical exam and electromyogram, a Trio, whole‐exome sequencing with mitochondrial genome sequencing, and deletion/duplication analysis of both nuclear and mitochondrial genomes were performed in December 2019 and revealed a *de novo*, pathogenic variant in C19orf12 [NM_001031726.3], c.256C>T (p.Q86X) located in exon 3. The methodology used in this analysis included next‐generation sequencing with copy number variant calling. The commercial laboratory has a mean depth of sequencing coverage of 145.41X for all three exons of *C19orf12* and there was no indication of a multi‐exon deletion/duplication involving this gene in the sequencing data. There were no additional variants identified during this analysis, including other well‐known genes associated with NBIAs.

Her most recent physical exam in October 2020 was notable for pale optic disc bilaterally, but normal social skills and cognition. The tone was normal in the upper extremities but markedly increased in bilateral ankles and knees at rest. She had ⅘ strength throughout. She had markedly increased tone with ankles plantarflexed at rest. Upon standing, the patient had to use her arms to pull to a stand, and for several minutes stood on the balls of her feet while bending at the waist. After putting pressure on the feet while making small ankle movements for approximately one to two minutes, she was able to ambulate. Gait is spastic with feet plantar flexed. Reflexes were 3+ at the patella, ankle, biceps, and triceps with a crossed adductor sign present. The sensation was intact to light touch and pinprick in all 4 extremities. No ataxia was present at this evaluation.

## 3 DISCUSSION

Our case report provides further evidence for the existence of MPAN as an inherited genetic disorder with both autosomal dominant and autosomal recessive modes of inheritance. This specific heterozygous variant, c.256C>T, discovered in our patient has previously been reported in a Subject 227 in Gregory et al. 2019. Both patients demonstrate a clinical presentation consistent with MPAN and the suggested autosomal dominant form of this condition. Subject 227 and our patient share a similar age of onset and clinical course, further providing evidence of the molecular mechanism outlined in both Monfrini et al. 2018 and Gregory et al. 2019. Table 1 summarizes all the clinical and genetic features of individuals with heterozygous pathogenic variants in *C19orf12* published in the medical literature (Gregory et al., 2019; Monfrini et al., 2018; Rickman et al., 2021). Interestingly, heterozygous pathogenic variants were reported in the original cohort of patients used to molecularly characterize MPAN in 2013, however, these individuals were theorized to have deep intronic variants or deletions in the opposite allele which were not detectable with available sequencing techniques (Hogarth et al., 2013). With both this patient's genetic testing results, the patient reported in Monfrini et al. in 2018, as well as the cohort of heterozygous pathogenic variants published by Gregory et al. 2019, the evidence is becoming abundantly clear that pathogenic variants in the *C19orf2* gene can lead to both autosomal dominant or recessively inherited forms of MPAN. The proposed dominant‐negative mechanism does provide a strong theory for pathogenesis as it would support the lack of difference in phenotype between the autosomal dominant and recessive forms, but functional studies are needed to further confirm this disease mechanism (Gregory et al., 2019).

**TABLE 1:** Heterozygous cases of MPAN in the medical literature

Publication	Genetic variant	Age (in publication)	Clinical summary
Current Report	c.256C>T (p.Gln86*)	17 years	Optic atrophy, pale optic disc, weakness in lower extremities, frequent falls, progressive dystonia, normal nerve conduction study, axonal motor neuropathy on electromyogram, hypertonia in ankles and knees, spastic gait
Monfrini et al., (2018)	c.265_266delAT (p.M89Gfs*12), *de novo*	High school aged	Language delay, IQ of 74, dysarthria, progressive imbalanced gait, right‐hand dystonia, precocious puberty, low vision, hypermetropia, astigmatism, optic atrophy, cervical dystonia, dysdiadochokinesia, tremor, lower limbs spastic hypertonia, patellar hyperreflexia, bilateral Babinski sign, substantia nigra and globus pallidus hypointensity, diffuse axonal motor neuropathy on electromyogram
Gregory et al., (2019)	c.227_237del11 (p.Met76 Thrfs*3)	55 years	Cognitive decline, parkinsonism
	c.227_237del11 (p.Met76 Thrfs*3)	37 years	Neuropsychiatric changes, progressive parkinsonism
	c.227_237del11 (p.Met76 Thrfs*3)	19 years	Neuropsychiatric changes, gait change, cognitive decline
	c.227_237del11 (p.Met76 Thrfs*3)	34 years	Gait imbalance, motor slowness, tremor, anxiety, progressive parkinsonism
	c.278delC (p.Pro93Leufs*26)	18 years	Optic atrophy, progressive parkinsonism, cognitive decline
	c.256C>T (p.Gln86*)	12 years	Gait changes, wheelchair at 18 years, optic atrophy, cognitive decline
	c.278dupC (p.Pro93Profs*8)	9 years	Cognitive decline, optic atrophy, dystonia, and dysarthria
	c.357dupG (p.Ala120Glyfs*32)	29 years	Neuropsychiatric changes, parkinsonism, cognitive decline
	c.279delT (p.Ala94Profs*25)	9 years	Falling, poor school performance, dysarthria
	c.300delT (p.Phe100Leufs*19)	5 years	Gait changes, optic atrophy, spastic paraparesis, cognitive decline
	c.268G>T (p.Glu90*)	22 years	Gait changes, depression, mild dystonia, dysarthria
	c.279_282delTGCC (p.Ala94Serfs*24), *de novo*	4 years	Developmental delay, spasticity, dystonia, disinhibited personality
	c.349C>T (p.Gln117*)	18 months	Dystonia, lower limb spasticity, sensorineural hearing loss
	c.238C>T (p.Gln80*)	10 years	Progressive spastic tetraparesis, optic disc pallor, dysphagia
	c.238C>T (p.Gln80*)	10 years	Progressive spastic tetraparesis, cognitive decline, optic disc pallor
	c.238C>T (p.Gln80*), *de novo*	5 years	Gait disturbance, optic atrophy, neuropsychiatric symptoms
	c.336G>A (p.Trp112*)	30 years	Neuropsychiatric symptoms, parkinsonism, dementia
	c.336G>A (p.Trp112*)	28 years	Cognitive decline, parkinsonism, dysarthria
Rickman et al., (2021)	c.278del G (p.Pro93Leufs*26), *de novo*	~37 years	Cognitive decline, gait instability, frequent falls, mood disturbances, slowed movements, ataxia, tremor, fine motor skill difficulties, non‐verbal at 37 years old, dystonic movements, upper and lower limb spasticity, hyperreflexia with bilateral extensor plantar responses, abnormal signal intensity in the globus pallidus

Note

Bold font indicates individuals with the same genetic variant are from the same family.

Notably, a limitation of our case report is the lack of updated brain imaging and the inaccessibility to the previous brain image, which hinders our ability to determine if our patient indeed has the characteristic iron accumulation of all NBIAs. Lastly, given that this pathogenic variant was *de novo*, we are unable to observe clinical features of MPAN segregating through the family that could lend support to a noticeable autosomal dominant inheritance pattern. Although the commercial lab indicates that the depth of coverage for this specific gene is high, there is always the possibility that a second undetectable variant by today's technology is present in the opposite allele and would indicate the genetic etiology in our patient is truly that of autosomal recessive MPAN.

From a clinical perspective, our findings further stress the variable inheritance pattern of MPAN and the importance of sending genetic testing for patients with clinical signs and symptoms consistent with MPAN. Heterozygous variants in *C19orf2* could be causative of a patient's MPAN‐like clinical presentation, therefore, it is important to critically evaluate any variant identified in *C19orf12* in this patient population. It is our hope that this publication and future publications regarding autosomal dominant MPAN will provide further support regarding disease pathogenesis, clinical presentation, and progression so that we can enhance patient care for individuals in this population.

## CONFLICTS OF INTEREST

The authors declare that they have no competing interests.

## AUTHOR CONTRIBUTIONS

Dr. Stuart Fraser wrote the original draft of the manuscript and performed a literature review. Kate Mowrey was the supervising faculty and provided edits for genetic nomenclature and checked the manuscript for accuracy. Kate Mowrey also made the included table. Dr. Mary Koenig and Dr. Laura Farach evaluated the subject in clinic and edited the manuscript for accuracy. Dr. Mancias identified the study subject, ordered the included genetic testing, and edited the manuscript for accuracy.

## Data Availability

Data sharing is not applicable to this article as no new data were created or analyzed in this study.
